# Design and test of powerful air-assisted sprayer for high stalk crops

**DOI:** 10.3389/fpls.2023.1266791

**Published:** 2023-11-03

**Authors:** Youyi Miao, Xiao Chen, Yan Gong, Dejiang Liu, Jian Chen, Guo Wang, Xiao Zhang

**Affiliations:** ^1^ Nanjing Institute of Agricultural Mechanization, Ministry of Agriculture and Rural Affairs, Nanjing, China; ^2^ Western Agricultural Research Center, Chinese Academy of Agricultural Sciences, Changji, China

**Keywords:** powerful air-assisted sprayer, high stalk crops, gas-liquid combined spraying, sprayer, wind-field distribution

## Abstract

The canopies of high stalk crops, such as maize, intersect the rows at the later stages of growth, making conventional sprayers unable to enter the field for spraying. Air-assisted sprayers are often used to improve the deposition of droplets inside the canopy. In this study, the sprayer structure, the air-assisted system, and the spraying system were designed. The air-assisted conveyor system characteristics were numerically analyzed, and the wind-field distribution was tested. The wind-field distribution results showed that the near-ground wind speed exceeded 5 m s^-1^ in the sampling interval from 10 to 35 metres. The wind field covered a concentrated spatial area with a downward pressure trend, resulting in better drift resistance and penetration. Field tests for droplet distribution were conducted at three maize heights to verify the powerful air-assisted sprayer's technical performance and working quality. The test results showed that the droplet deposition and coverage decreased gradually along the range direction, and the top layer had the highest deposition and coverage across the canopy. The upper canopy of 0 to 12 metres range demonstrated a greater extent of coverage and deposition. The peak deposition area expanded from 9 to 33 metres in the lower canopy, with an average value of 3.77 μg cm^-2^. The droplet coverage within the 30 to 60 metres range only amounted to 15% to 18% of the total coverage.

## 1 Introduction

Maize planting area in China has reached 43,324 thousand hectares, constituting 25.7% of the total crop planting area in 2021. Maize canopies intersect the rows in the later stages of growth, making conventional sprayers unable to enter the field for spraying operations. Once encountering aggressive pests and diseases, it is often difficult to effectively control, leading to a significant reduction in maize yields or even a failure of the harvest. This poses a serious threat to China’s food security. Fall armyworm invaded twenty-six domestic provinces in 2019, threatening over 50% of the crop planting area in China (Yang et al., 2019; Wang and Lu, 2020). The timing of pest control is critical. Once a pest infestation is identified, all spraying operations must be completed in a very short time (Wang et al., 2014; Kumar et al., 2021).

Air-assisted spray is an advanced application technology recommended by the Food and Agriculture Organization of the United Nations (FAO) (Czaczyk, 2012; Gu et al., 2022). In agricultural pest control, pneumatic sprayers equipped with large axial-flow or centrifugal fans have been widely used in developed countries (Khot et al., 2012; Sinha et al., 2019). Hong et al. (2018) designed an air-assisted sprayer that integrated air-assisted, variable spraying, and intelligent targeting technologies, which could be used for pests and diseases of fruit trees with different canopy shapes. Thakare et al. (2015) evaluated an air-assisted sleeve boom sprayer machine and achieved effective pest control.

Derksen et al. (2008) utilized the Jacto air-assist sprayer equipped with JA3 hollow-cone nozzles in soybean canopy. This specific method generated the highest concentration accumulation of fungicide residues on leaves in the lower part of the canopy. The Italian company TIFONE has developed a series of wind-driven long-range sprayers for maize, soybeans, and other crops. These sprayers have a maximum range of 30 metres and are designed with horizontal inflow ducts, similar to wind-driven sprayers used in orchards.

In recent years, Chinese scholars have made significant advancements in orchard wind-delivery technology and equipment, focusing on enhancing efficiency and reducing volume spraying. Li et al. (2021) designed and constructed an air-fed sprayer equipped with an axial fan and annular nozzle. The dimensions and placement of the nozzle were determined through computational fluid dynamics (CFD) fluid simulation. Zhou et al. (2015) developed an air-assisted electrostatic sprayer combined with air-assisted spraying and electrostatic spraying technology. With the development of Unmanned Aerial Vehicle (UAV), field crop spraying by plant protection UAV was widely accepted (Qin et al., 2023). Hussain et al. (2022) compared the spraying effect of different HBL dosages and sprayer volumes of KMS (Knapsack manual sprayers) and UAV sprayers on maize crop growth and development. The results showed that the droplet deposition of UAV (15 30 L ha^-1^) was higher than KMS; the average deposition was between 0.05 and 0.06 μL cm^-2^. However, the UAV sprayer had a poor droplet coverage rate, which was below 10%. The low coverage results were similar to Sarri et al. (2019). The plant protection UAV equipment has the features of mobile flexibility and high operational efficiency. However, the protection effect for high stalk crops still needs to be improved due to poor penetration and extremely small number of droplets (Abd. Kharim et al., 2019; Guo et al., 2020; Zhan et al., 2022; Chang et al., 2023).

Studies have shown that the effective deposition of droplets inside the canopy can be improved by the air-assisted spray system. However, there is a lack of research and application of wind-delivered application technology and ground equipment for field crops in China. Wang et al. (2021) designed a crawler self-propelled corn interrow sprayer that could meet the space requirements for plant protection operations under the narrow row of corn leaves below 600 mm. The high clearance boom sprayer with an air-assisted system is commonly employed for maize crops due to its good spray uniformity and control effects. However, the equipment will be invalid when the height of the maize exceeds the ground clearance of the equipment (Wang et al., 2015; Wei et al., 2016; Wu et al., 2018).

In this study, a powerful air-assisted remote sprayer was developed to overcome the challenges of mechanized plant protection for maize and other crops in China. The wind field distribution characteristics were numerically analyzed, and wind field distribution tests and prototype droplet deposition distribution tests were conducted in maize fields to provide a new type of application technology and equipment that is economical and efficient for pest and disease control in this crop.

## 2 Materials and methods

### 2.1 Structural composition and working principle

**Figure 1:**
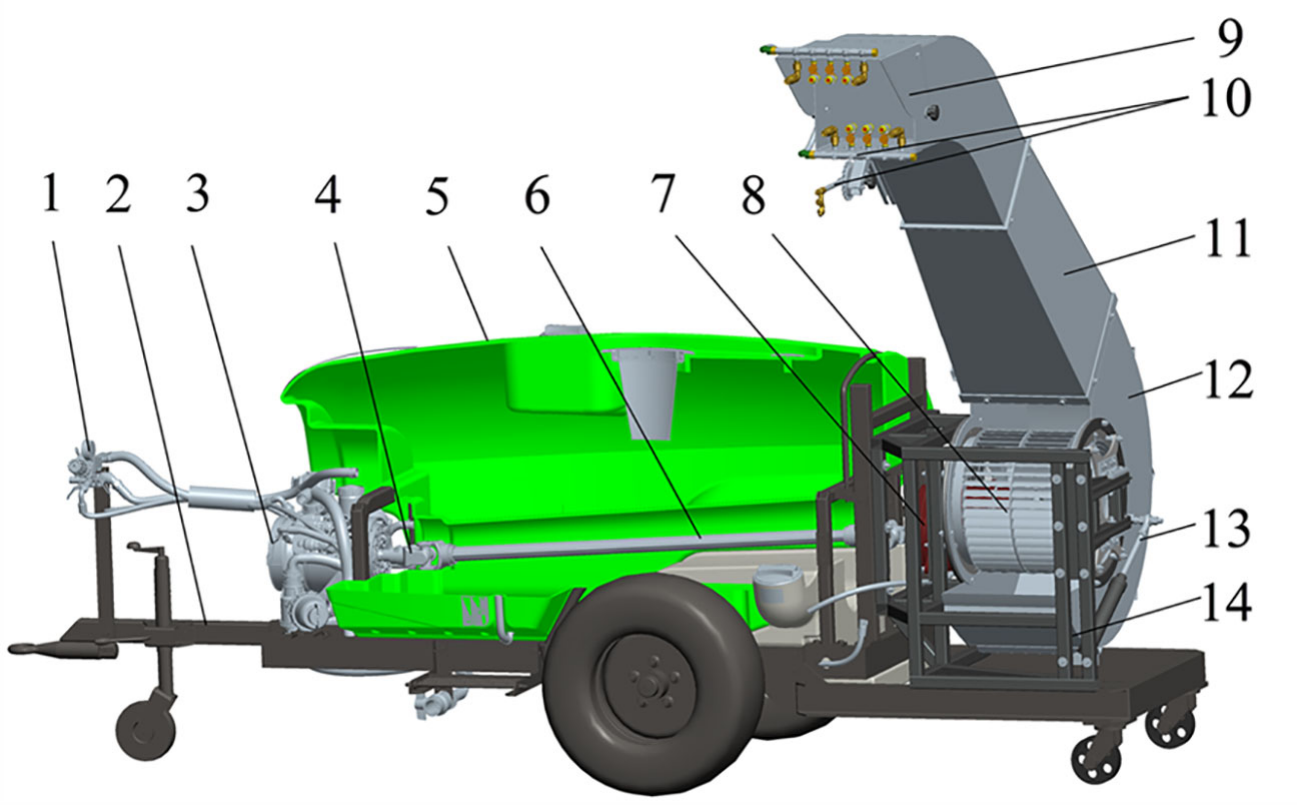
Structure of powerful air-assisted sprayer. 1. distribution valve 2. tractor frame 3. diaphragm pump 4. universal joint 5. pesticide tank 6. drive shaft 7. gearbox 8. multi-wing centrifugal fan 9. deflector cap 10. spray components 11. deflector duct 12. turbine casing 13. turbine casing rotating mechanism 14. frame.

The powerful air-assisted sprayer structure consists of a spraying system, an air-assisted conveyor system, a power transmission system, and a traction frame, as is shown in **Figure 1**. The spraying system is composed of a diaphragm pump (3), a distribution valve (1), a pesticide tank (5), and spray components (10). The air-assisted conveyor system is comprised of a multi-wing centrifugal fan (8), a turbine casing (12), a deflector duct (11), a deflector cap (9), a turbine casing rotating mechanism (13) and a frame (14). The power transmission system is composed of a universal joint (4), a drive shaft (6), and a gearbox (7). The gearbox (7) is connected to the output shaft of the diaphragm pump (3) through the universal joint (4). The multi-wing centrifugal fan (8) is mounted on the gearbox (7). The powerful air-assisted sprayer is connected to the tractor through the tractor frame (2), and the tractor power output shaft links to the input shaft of the diaphragm pump (3). The turbine casing rotating mechanism (13) is driven by the hydraulic system of tractor.

The powerful air-assisted sprayer is towed and powered by a tractor and can be used on field roads. The air-assisted conveyor system has the capability to generate secondary atomization while pushing droplets to a longer distance, significantly enhancing the delivery range and penetration of the droplets.

### 2.2 Design of the air-assisted conveyor system

The air-assist conveyor system is the core technology of powerful air-assisted sprayer. Its working performance directly impacts the conveyance distance and the penetration capacity of droplets. The air-assisted conveyor system for plant protection in high stalk crops requires higher air volumes, faster speeds, and uniform airflow direction compared to orchard air-assist sprayers.

#### 2.2.1 Overall structure of air-assisted deflector duct

**Figure 2:**
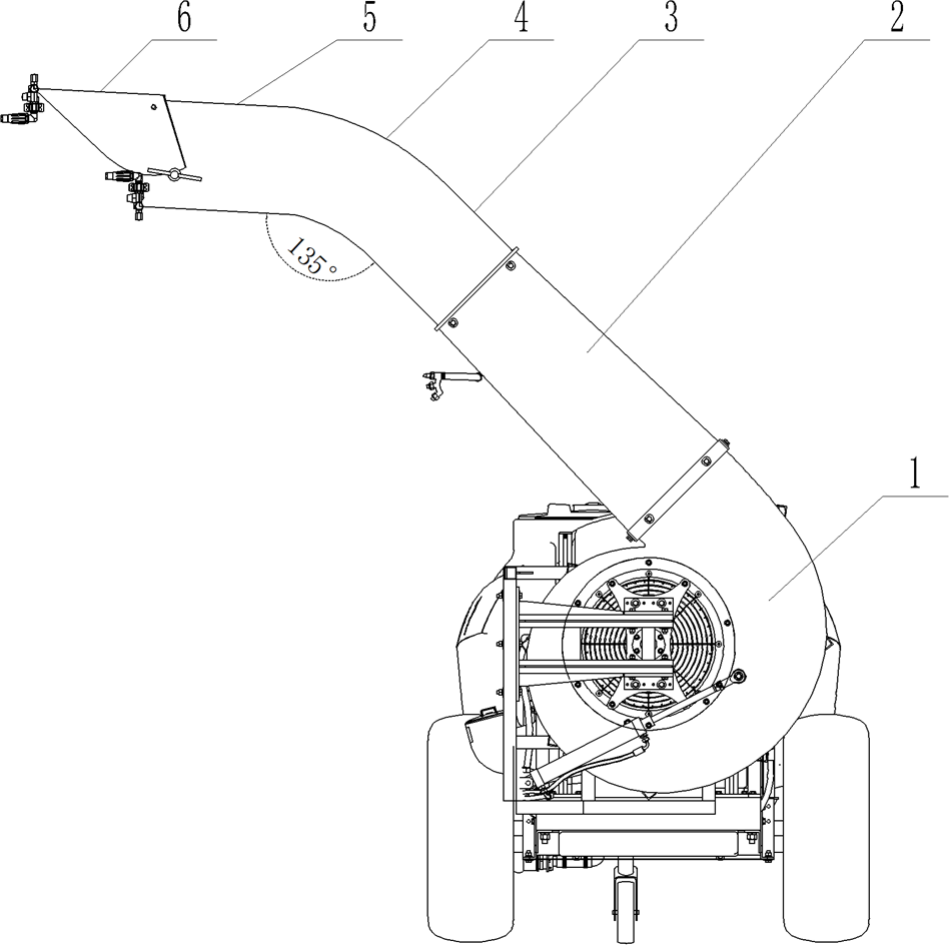
Structure of air-assisted deflector duct. 1. turbine casing 2. contraction section 3. straight section A 4. arc section 5. straight section B 6. deflector shield.

The structure of the air-assisted deflector duct is shown in **Figure 2**, which is composed of turbine casing (1), contraction section (2), straight section A (3), arc section (4), straight section B (5), and deflector shield (6). The contraction section (2) is a trapezoidal structure that further augments the velocity and pressure of the wind. The arc section (4) connects straight section A (3) and straight section B (5), forming an angle of 135°. This design not only redirects the flow field but also minimizes wind energy loss effectively.

The deflector shield (6) is set in the upper part of the air outlet of the deflector duct, and the angle can be adjusted from 0° to -5°. The adjustable angle of the deflector shield can also meet the varying requirements of different meteorological conditions in the field for spraying. This feature allows the shield to effectively mitigate the adverse effects of air movement in the environment, reducing the drift of insecticide droplets to non-target areas. By adjusting the angle of the deflector shield to form a downward airflow, it is possible to enhance the dispersion of pesticide droplets towards the target crop, particularly in the lower areas.

**Figure 3:**
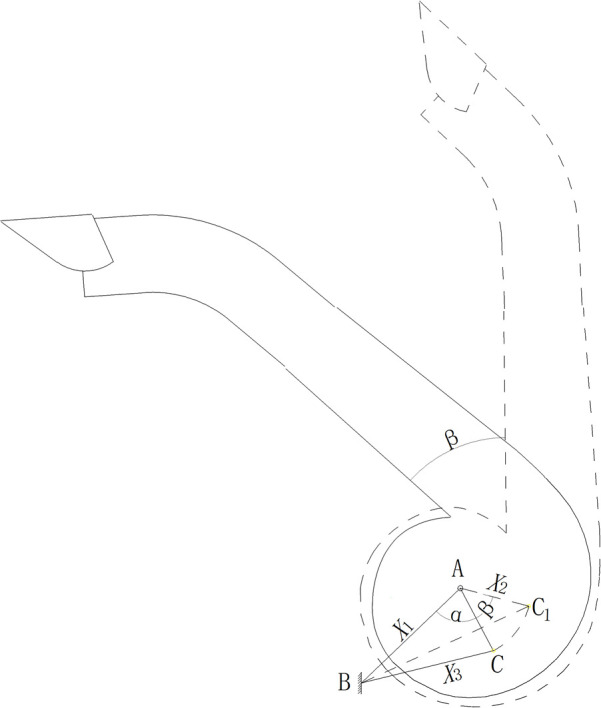
Diagram of the steering mechanism.

**Figure 3** illustrates the steering mechanism motion sketches of deflector duct. The turbine casing is designed to coincide with the fixed-ring and fan axis, ensuring no alteration to the airflow in the turbine casing or deflector duct. This ensures that the application equipment maintains spraying consistency and stability without wasting extra wind energy.

Equation (1) shows the correlation between the telescopic length of the cylinder and the rotation angle of the inflow air cylinder.


(1) $$X_{3}=\sqrt{{\text{X}}_{1}^{\;2}+{\text{X}}_{2}^{\;2}-2{\text{X}}_{1}*{\text{X}}_{2}*\text{cos}(\text{α}+\text{β})}$$

Where *X*_1_ is the distance from fan axis A to hinge point B of hydraulic cylinder on frame, mm; *X*_2_ is the distance from fan axis A to hinge point C of hydraulic cylinder on turbine casing, mm; *X*_3_ is the hydraulic cylinder telescopic length, mm; *α* is the initial angle of deflector duct, °; *β* is the adjustable angle of deflector duct, °.

The designed parameter of *α* is 74°, *X*_1_ is 1136 mm, and *X*_2_ is 586 mm. Inserting the known values obtains a result of hydraulic cylinder telescopic length range from 1126 to 1526 mm, and 47° adjustable angle of deflector duct.

#### 2.2.2 Design of turbine casing steering mechanism

To avoid uneven stresses and deformations in the structure of the air-assisted system caused by the heavy weight of deflector duct, the turbine casing is constructed with the double support structure.

**Figure 4:**
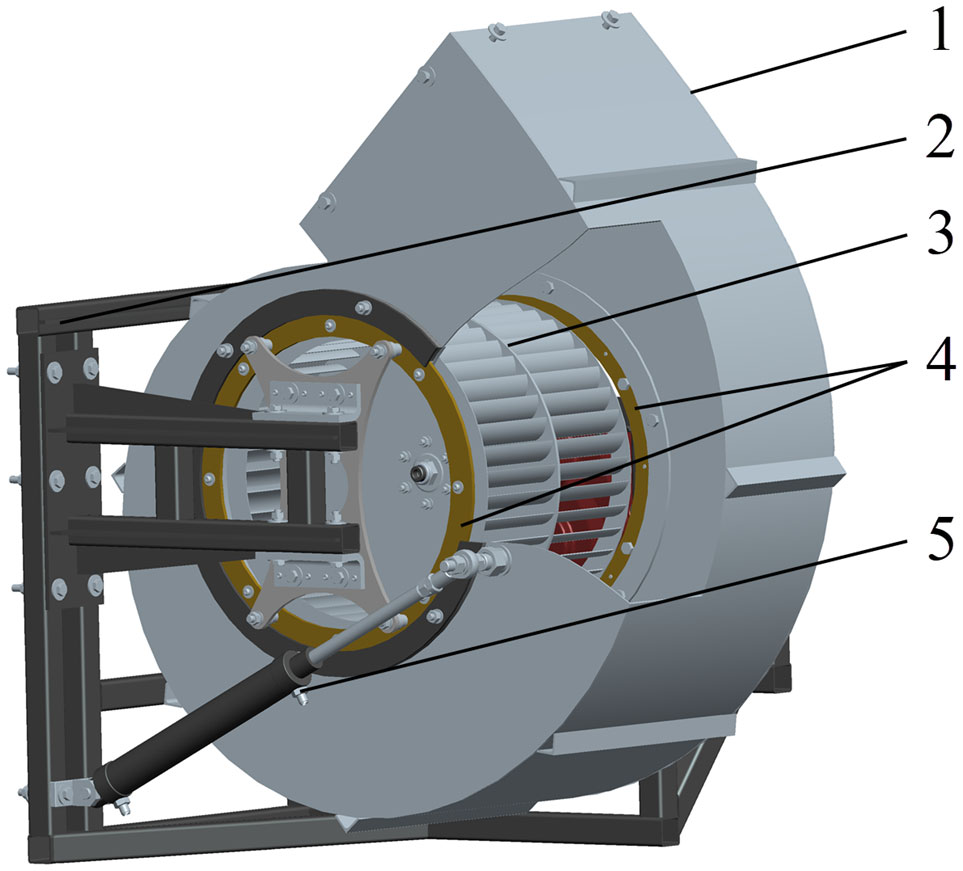
Structure of the turbine casing steering mechanism. 1. turbine casing 2. frame 3. fan 4. fixed-ring 5. hydraulic cylinder.

As shown in **Figure 4**, the turbine casing (1) coincides with the fixed-ring (4) and fan (3) axis; the hydraulic cylinder (5) is hinged on the frame (2) at one end and on the turbine casing (1) at the other end. By adjusting the telescopic length of the hydraulic cylinder (5), the turbine casing (1) (together with the deflector duct) is driven to revolve on the flange of fixed-ring (4), thus changing the spray angle at the outlet of conveyor system.

#### 2.2.3 Calculation of air volume of powerful air-assisted system

The design of the fan and the calculation of the air volume in the powerful air-assisted system are mainly based on the replacement principle and the end velocity principle (Dai, 2008; Ru et al., 2022). The main function of the designed air-assisted system is to transport the airflow from the fan to the far side. The replacement principle’s space volume should be the area between the sprayer outlet and the top of the crop canopy.

**Figure 5:**
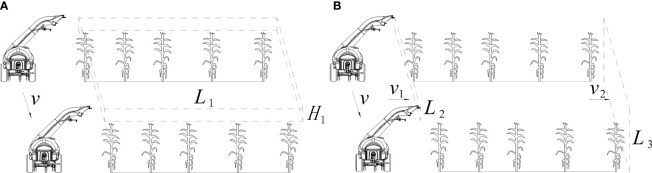
Calculation principle of powerful air-assisted system. **(A)** Replacement principle. **(B)** End speed principle.

As shown in **Figure 5A**, according to the replacement principle, with the sprayer’s driving speed and the fan’s constant rotation speed, the air volume generated by fan per second is equal to the volume of the rectangular.

The calculation of the required air volume is given by Equation (2).


(2) $${\text{Q}}_{1}=L_{1}H_{1}\nu K_{1}$$

Where *Q*_1_ is the air volume generated by ducts, m^3^ s^-1^; *L*_1_ is the range of sprayer, m; *H*_1_ is the height between the sprayer outlet and the top of crops, m; *v* is the driving speed of sprayer, m s^-1^; *K*_1_ is the coefficient of air attenuation and loss, *K*_1_ = 1.3~1.6. Although the horizontal direction wind loss of the outlet is small, the fan under pressure wind will be blown into the crop inside, taking *L*_1_ = 50 m, *K*_1_ = 1.4. According to Equation (2), the required air volume *Q*_1_ is 7 m^3^ s^-1^.

As shown in **Figure 5B**, according to the end velocity principle, the airflow must keep a certain velocity when it reaches the end of its range. This ensures that crop leaves could be flipped by the airflow at a distance in the direction of the shot to improve droplet penetration and adhesion.

The initial velocity must satisfy the following Equation(3).


(3) $$L_{2}\nu_{1}\geq L_{3}\nu_{2}K_{2}$$

Equation(3) can be used to obtain:


(4) $${\text{ν}}_{1}\geq \frac{L_{3}\nu_{2}K_{2}}{L_{2}}$$

Where *v*_1_ is the initial velocity, m s^-1^; *v*_2_ is the end velocity, m s^-1^, *v*_2_ = 2∼4 m s^-1^; *L*_2_ is the length of duct outlet, m; *L*_3_ is the length at the end of the range area, m; *K*_2_ is the coefficient of air resistance, *K*_2_ = 1.3~1.8.

According to the design parameter and cultivation requirement of crops, take *L*_3_ = 3 m, *v*_2_ = 3 m s^-1^, *L*_2_ = 0.35 m. Considering the resistance against the airflow because of dense crop canopy in late growth period, take K_2_ = 1.8. According to the Equation (4), the required initial velocity *v*_1_ is not less than 46 m s^-1^.

#### 2.2.4 Design of multi-wing centrifugal fan

The designed multi-wing centrifugal fan with double inlet must satisfy the flow rate *Q*_1_≥7 m^3^ s^-1^, *v*_1_≥46 m s^-1^. The full pressure of the fan mainly consists of the dynamic pressure loss and static pressure loss (friction pressure loss and local pressure loss), which is calculated as Equation (5).


(5) $$\left\{ \begin{array}{c} P_{\text{d}}=\frac{1}{2}\rho v_{1}^{2}\\ P_{\text{f}}=\lambda \frac{\rho v_{1}^{2}}{2D}L\\ P_{\text{l}}=\xi \frac{1}{2}\rho v_{1}^{2}\\ P_{\text{tF}}=P_{\text{d}}+P_{\text{f}}+P_{\text{l}} \end{array}\right.$$

Where *P*_d_ is the dynamic pressure loss, Pa; *P*_f_ is the friction pressure loss, Pa; *P*_l_ is the local pressure loss, Pa; *P*_tF_ is the full pressure, Pa; *ρ* is air density, kg m^-3^; *λ* is the friction coefficient; *D* is the equivalent diameter, m; *L* is the length of deflector duct, m; *ξ* is the local resistance coefficient.

The selected values of each parameter are: *ρ*=1.21 kg m^-3^, *λ*=0.18, *D*=0.44 m, *L*=2.46 m, *ξ*=0.31, According to Equation (5), the full pressure *P*_tF_ is 2965 Pa.

The designed impeller rotation speed is 2200 r min^-1^. The specific speed can be calculated by Equation (6).


(6) $$n_{s}=5.54n\frac{{\left(\frac{Q_{1}}{2}\right)}^{1/2}}{p_{\text{tF}}^{\;3/4}}$$

Where *n*_s_ is the specific speed; *n* is the impeller rotation speed, r min^-1^.

The calculated specific speed of multi-wing centrifugal fan is 56.75, which belongs to the range of forward-bladed impeller centrifugal fans. **Table 1** shows the main structural parameters of multi-wing centrifugal fan.

**Table 1:** Basic structural parameters of multi-wing centrifugal fan.

Parameters	value
Inner diameter of impeller *D*_1_/mm	360
Outer diameter of impeller *D*_2_/mm	450
Number of blades *z*/pcs	42
Impeller width *b*/mm	324
Turbine casing width *B*/mm	400
Blade inlet angle *β*_1A_/°	69
Blade outlet angle *β*_2A_/°	131

#### 2.2.5 Simulation of the air-assisted conveyor system

The numerical calculation was carried out for the air-assisted conveyor system by Fluent. To improve the accuracy of the simulation results and computational efficiency, mesh refinement was performed on centrifugal fan. The volume was meshed with poly-hexcore body, size from 2 mm to 20 mm. The total number of elements was 3,080,962.

The control equations were Navier-Stokes equations, the turbulence was calculated by Realizable k-ϵ model. The near-wall equations were in standard wall function, The pressure-velocity coupling was in Coupled algorithm, and the pressure discrete format was in PRESTO! Format. The momentum, energy and turbulence dissipation equations were in second-order windward format, and the computational convergence residuals were set to 0.0001. The inlet and outlet were given pressure inlet and pressure outlet boundary conditions, and the value was set to zero during simulation experiments. The impeller area was set as a rotating area, and the Frame Motion model was used to set the rotating area speed at 2200 r min^-1^.

### 2.3 Design of the spraying system

**Figure 6:**
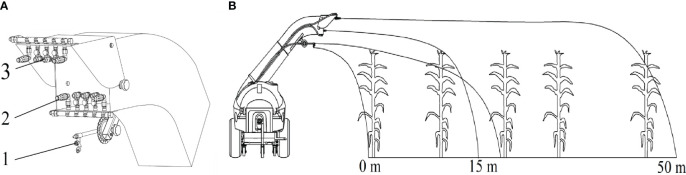
Working principle of the spraying system. **(A)** Structure of spraying system: 1. multi-heads hydraulic nozzle 2. high-pressure long-shot nozzle 3. cone nozzle. **(B)** Distribution of spraying range.

To achieve uniform distribution of pesticide droplets in the full spray range, the nozzle combination was designed with multi-heads hydraulic nozzle, high-pressure long-shot nozzle, and cone nozzle (**Figure 6A**). As shown in **Figure 6B**, in the range from 0 to 15 metres, the airflow has not yet deposited in the region, this area of the crop using multi-heads hydraulic nozzle spraying method. The multi-heads hydraulic nozzle is mounted on the out wall of the deflector duct, and the installation height from the ground is 1.5 metres. In the range from 15 to 50 metres, using the air-assisted method to transport the droplets to this interval. The high-pressure long-shot nozzle and cone nozzle are distributed on the upper and lower sides of the deflector shield air outlet. The strong airflow generated by the fan makes two kinds of nozzles spraying pesticide droplets to remote distribution, effectively covering target crops in the deposition area. The parameter of spraying system is shown in **Table 2**.

**Table 2:** Technical parameter of spraying system.

Parameters	Quantity	Working pressure/MPa	Flow rate/L min^-1^
Multi-heads hydraulic nozzle	1	4.0	19
High-pressure long-shot nozzle	4	4.0	8.4
Cone nozzle	6	4.0	6.5

### 2.4 Experimental design and methods

#### 2.4.1 Design and measurement of the wind-field distribution test

**Figure 7:**
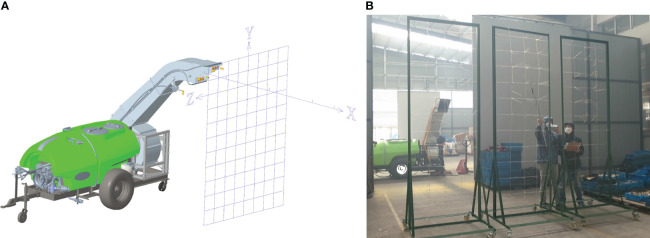
Wind-field distribution measurement method. **(A)** Sampling grid frame. **(B)** Wind-field distribution test.

The wind-field distribution test was conducted in the wind-filed lab of XINYI Agricultural Machine Co. Ltd, Taizhou, Zhejiang Province. A tractor of 100 HP was applied to drive the centrifugal fan. The sampling grid frame was a rectangle frame with 2750 mm×3000 mm, divided into several grids of 250 mm×250 mm (**Figure 7A**). Before testing, a cartesian coordinate was set with the center of the fan outlet as the wind measurement origin, the horizontal direction as the X-axis and the vertical direction as the Y-axis.

The sprayer was located stably on the ground, and the fan outlet was adjusted to a horizontal status by rotating the hydraulic cylinder. The sampling grid frame was located vertically to the X-axis. The wind speed value at each node was measured by KA33 thermal sphere anemometer, and each node was measured in turn from the center to the surrounding area until the measured wind speed value was less than 2 m s^-1^. The sampling grid frame was moved at intervals of 5 metres along the range direction, and the wind speed values at each node at the corresponding position were measured separately (**Figure 7B**).

#### 2.4.2 Design of spray field test

**Figure 8:**
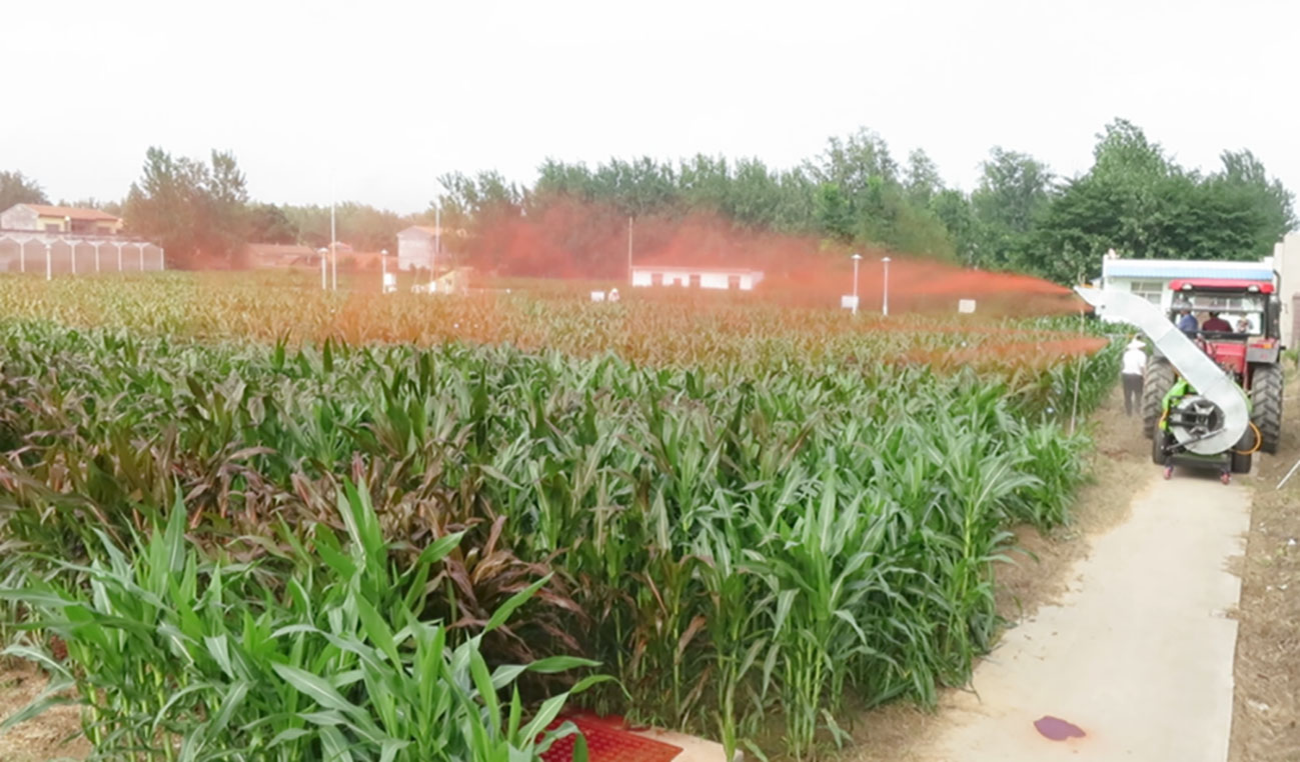
Field test.

The spray field test was conducted in maize planting base of the Institute of Farmland Irrigation of CAAS, Shangqiu, Henan Province (**Figure 8**). The maize row spacing was 0.4 metres and the plant spacing was 0.5 metres. Three trials were conducted at different growth height of maize at 1.4, 1.7 and 2 metres named as TM1, TM2 and TM3. The sprayer was towed by a 100 hp tractor and operated at travel speeds of 3.6 km h^-1^.

The fluorescent tracer dye, Allura Red was used as spray tracer to verify the droplet deposition and coverage performance. The tracer was dissolved in water at about 5 g L^-1^. The filter paper (Φ90 mm) and water-sensitive paper (26 mm×76 mm, Syngenta) were applied to collect the spray droplets. The visible spectrophotometer (V5100, Developed Shanghai METASH Instruments Co., Ltd) and the droplet analysis software of DepositScan (Developed by USDA) were applied to determine and analyze droplet deposition and coverage on filter paper and water-sensitive paper.

In this study, according to the maize cultivation spacing, twenty-one plants of maize with an interval of 3 metres each were selected as an experimental row along the spray range. Along the travel direction of sprayer, three same experimental rows as mentioned above with an interval of 5 metres were selected. For TM1, three layers of filter paper were fixed on the leaves from the top leaf with an interval of 50 cm. For TM2 and TM3, four layers of filter paper were fixed on the leaves from the top leaf with an interval of 50 cm. Meanwhile, beside each filter paper, one piece of water-sensitive paper was fixed for each layer. After spraying was completed, the filter paper and water-sensitive paper were collected (in less than 10 min) and placed in resealable plastic bags.

### 2.5 Data analysis

#### 2.5.1 The determination of droplet deposition

The filter paper was placed individually into a glassware filled with 20 mL of water and kept soaking for 3~4 hours to make sure that the Allura Red was completely eluted. A certain amount of liquid was pipetted into the glass cuvette, and placed into the spectrophotometer to measure the absorbance of the liquid. According to Equation (7), the absorbance of the liquid was converted to the value of droplet deposition on the filter paper.


(7) $$p=\frac{\rho_{\left(A\right)}\times V}{S}=\frac{(24.83\times A+0.031)\times V}{S}$$

Where *p* is the droplet deposition value, μg cm^-2^; *ρ_(A)_* is the mass concentration of washed-out Allura Red, mg L^-1^; *A* is the absorbance of Allura Red liquid; *V* is the volume of water used to soak the filter paper, mL; *S* is the area of filter paper, cm^2^.

#### 2.5.2 The determination of droplet coverage

The parameters of droplet size, droplet number and droplet coverage on water-sensitive paper were analyzed by the image processing software DepositScan. The recycled water-sensitive paper was scanned in grayscale mode with a resolution of 600 DPI, and then the scanned images were imported into DepositScan to analyze the droplet coverage.

## 3 Results and discussion

### 3.1 Simulation test

**Figure 9:**
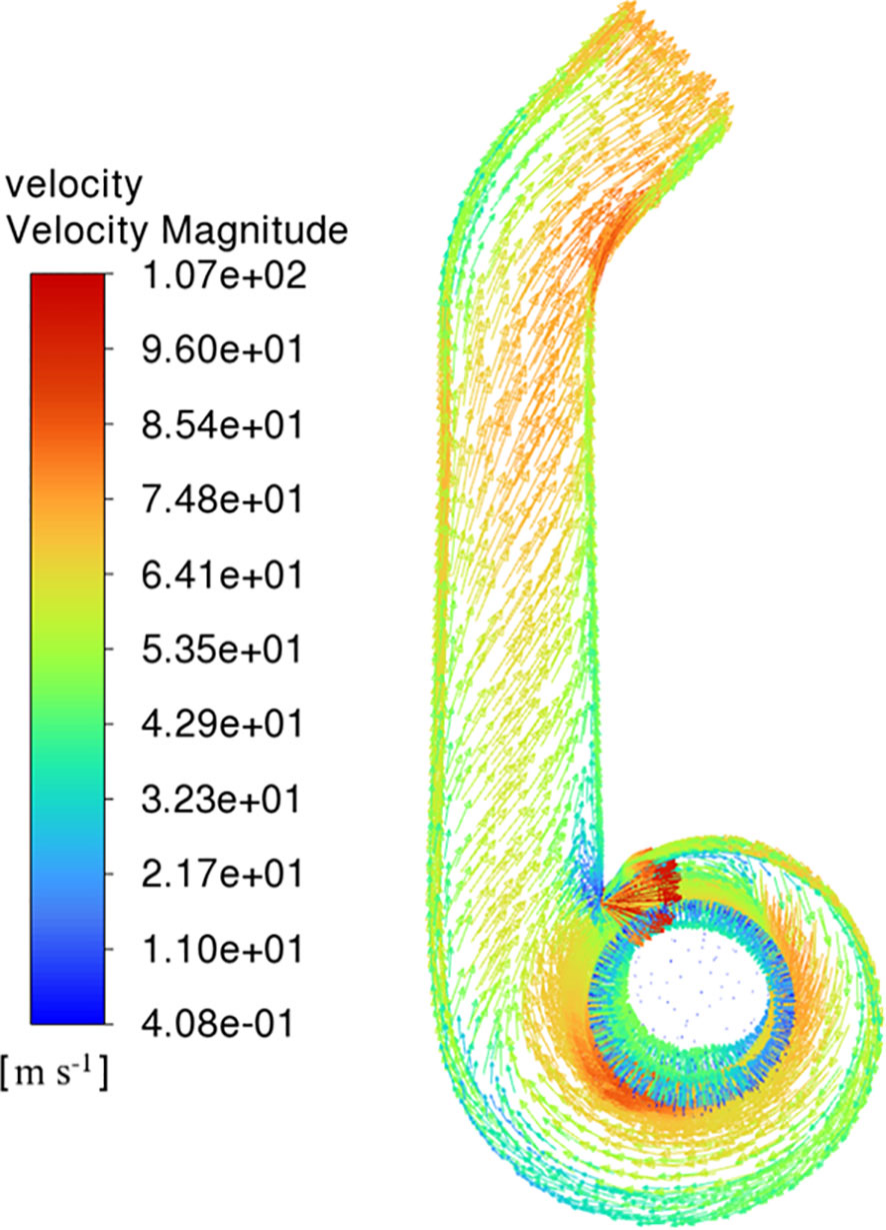
Contour of velocity.

As shown in **Figure 9**, the velocity distribution inside the deflector duct is evenly distributed. Due to the influence of airflow space, the wind speed on the impeller surface is higher in the area close to the turbine casing outlet than in the narrow area of the turbine casing. The cross-section gradually contracts, and the wind speed gradually increases after the airflow enters the contraction section. The wind speed on the outside of the arc section is lower than the wind speed on the inside because of air flow is blocked by the wall. After the airflow is guided to the outlet of deflector duct, the airflow direction is consistent with the outlet direction, leading to a decrease in overall turbulence. The average wind speed at the outlet reaches 65.3 m s^-1^, and the outlet air volume flow rate is 7.85 m^3^ s^-1^.

**Figure 10:**
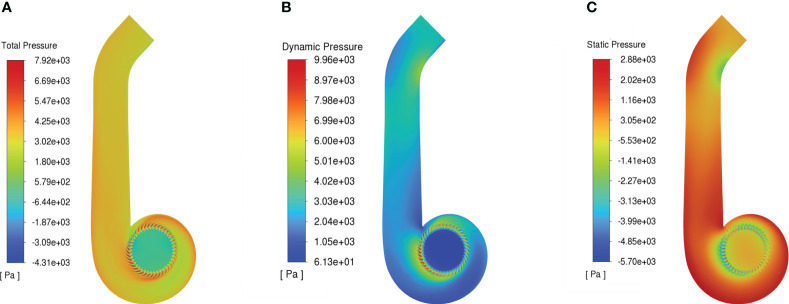
Contour of pressure. **(A)** Total pressure. **(B)** Dynamic pressure. **(C)** Static pressure.

**Figure 10** shows the pressure distribution of air-assisted deflector duct. The full pressure of the deflector duct is well distributed and the average full pressure at the outlet is 3200 Pa (**Figure 10A**). Dynamic pressure is the main contributor to the total pressure in the deflector duct. The dynamic pressure is higher around the impeller and at the outlet, and the trend is consistent with the airflow velocity field distribution (**Figure 10B**). The static pressure increases gradually in the direction of the impeller diameter, with the highest pressure at the wall of turbine casing and contraction section, and then decreases gradually in the direction of the outlet (**Figure 10C**).

### 3.2 Test of wind-field distribution

**Figure 11:**
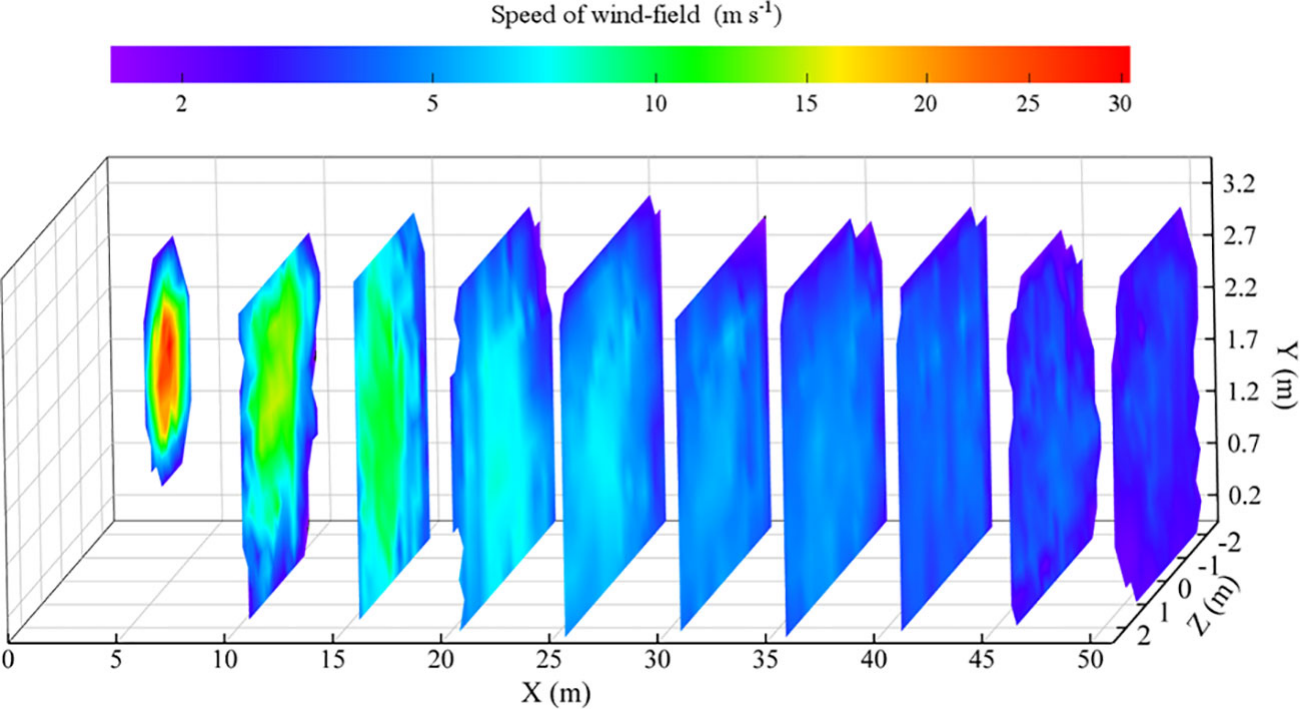
Cloud map of wind-field distribution.

The wind-field distribution results were shown in **Figure 11**, the wind-field range of the sampling grid frame at 5 m from the air outlet was concentrated, and the maximum wind speed of the cross section was large than 30 m s^-1^. With the increase of the distance from the outlet, the wind-field of the sampling plane was expanded gradually, and at the 10 metres sampling position, the lower boundary of the wind-field was expanded to the ground, while the upper boundary of the wind field was not significantly raised. The near-ground wind speed exceeded 5 m s^-1^ in the sampling interval from 10 to 35 metres, which showed the downward pressure effect of the wind-field. When the sampling distance reached 40 metres, the maximum wind speed area gradually disappeared, and the overall wind speed of the sampling plane became stable, with an average wind speed of 3.8 m s^-1^. The wind speed further decreased at 50 metres position, but the average wind speed at the node in the sampling grid frame still met the effective wind speed of more than 2 m s^-1^.

### 3.3 Field test

#### 3.3.1 Distribution of droplet deposition

**Figure 12:**
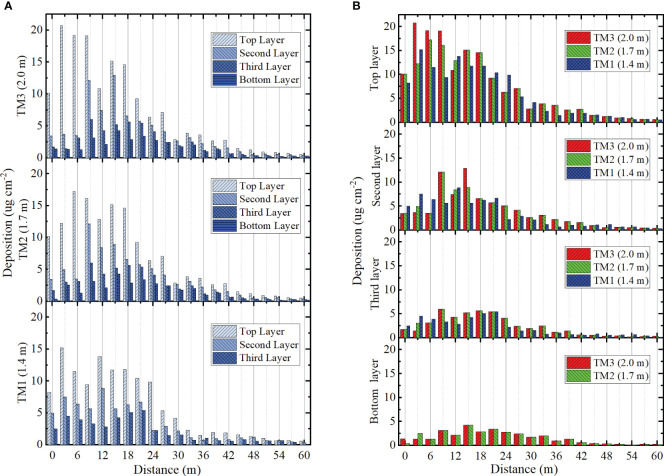
The deposition distribution of canopy. **(A)** Deposition distribution of three trials. **(B)** Deposition distribution in four layers.

The distribution of droplet deposition for three trials was shown in **Figure 12**. In **Figure 12A**, there was a clear trend of decreasing in droplet deposition along the direction of range for all three trials. The TM1 trial had an average deposition of 5.9 μg cm^-2^ in the top layer, 3.2 μg cm^-2^ in the second layer and 2.1 μg cm^-2^ in the third layer. A reduction of 45.7% in the second layer and a reduction of 64.4% in the third layer compared to the first layer. The highest deposition across the canopy was in the top layer. TM3 had the highest deposition and TM1 had the lowest deposition in the top layer of the three trials. This indicated that there was a positive correlation between deposition levels and proximity to the outlet. The droplet deposition was significantly higher in the top layer compared to the other layers within the 0 to 6 metres range.

In the multi-heads hydraulic nozzle range of 0 to 15 metres, the deposition in the top layer of TM1, TM2, and TM1 was 15.7 μg cm^-2^, 14.1 μg cm^-2^, and 11.7 μg cm^-2^, respectively (**Figure 12B**). This was more than three times the average value of the layer in which it was located. In the second layer, the area of peak deposition concentration was observed within the range of 9 to 21 metres. In the third layer, the area of peak deposition extended from 9 to 33 metres, exhibiting an average value of 3.77 μg cm^-2^. This indicated that as the range expanded, deposition in the lower and middle layers of the crop distributed further, reflecting the advantages of a downward-pressure wind field. In the bottom layer, droplet deposition could still be presented in TM2 and TM3 with a mean value of 1.59 μg cm^-2^ and 1.61 μg cm^-2^. The well-distributed deposition indicated the effective penetration of the wind field.

#### 3.3.2 Distribution of droplet coverage

**Figure 13:**
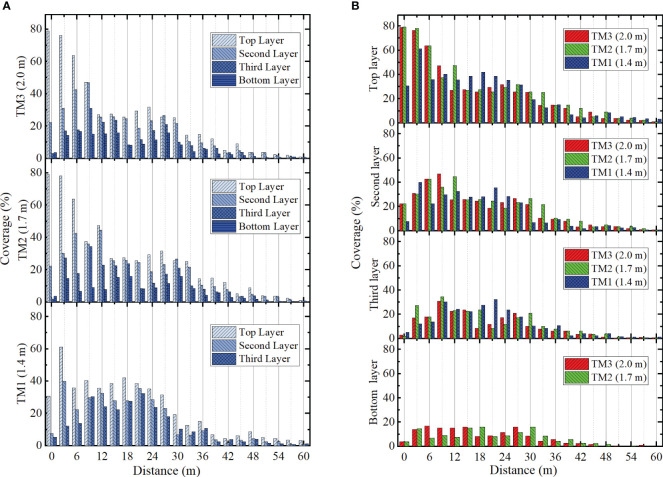
The coverage distribution of canopy. **(A)** Coverage distribution of three trials. **(B)** Coverage distribution in four layers.

The distribution of droplet coverage for three trials was shown in **Figure 13**. The average coverage of TM1 was 22.8% in the top layer, 15.2% in the second layer and 12.3% in the third layer (**Figure 13A**). The average coverage of TM2 was 26.9% in the top layer, 18.5% in the second layer, 12.0% in the third layer and 6.9% in the bottom layer. The average coverage of TM3 was 25.1% in the top layer, 17.0% in the second layer, 10.3% in the third layer and 7.3% in the bottom layer. TM2 and TM3 exhibited superior coverage than TM1. TM2 demonstrated better coverage than TM3 in the initial three layers and had inferior coverage in the bottom layer. In the range of 0 to 30 metres, the average coverage of TM1, TM2, and TM3 in the top three layers was measured as 28.9%, 30.4%, and 27.6% respectively. Conversely, in the range of 30 to 60 metres, the average coverage of TM1, TM2, and TM3 in the top three layers was measured as only 4.9%, 6.7%, and 4.8% respectively. Although the three trials satisfied the design requirements for droplet coverage within the 30 to 60 metres range, the spray coverage achieved only amounted to 15% to 18% of the total coverage.

In the top layer, TM1 exhibited more extensive coverage than TM2 and TM3 within the 12 to 24 metres range (**Figure 13B**). After 24 metres, the trend was consistent with TM2 and TM3. In the second layer, the coverage of the three trails exhibited consistency with the top layer from 12 to 24 metres. TM2 and TM3 maintained their dominance in the 24 to 42 metres range. In the third layer, the coverage of three trials experienced a decrease to 5% after 39 metres. In the bottom layer, the distribution of TM2 and TM3 coverage exhibited greater uniformity and still reached 2% at 45 metres range.

## 4 Conclusions

1. In view of the difficulty of plant spraying after the canopies cross the rows of maize and other crops, a powerful air-assisted remote sprayer which could be sprayed on the field road was designed. The adjustable air-assisted conveyor system and combined gas-liquid remote uniform spraying system were designed to achieve uniform and effective droplet coverage over the entire spraying area.
2. The wind-field test results showed that the wind field could reach more than 50 metres. The near-ground wind speed exceeded 5 m s^-1^ within the sampling interval from 10 to 35 metres. The wind field covered a concentrated spatial area and had a downward pressure trend, resulting in better drift resistance and penetration, which helped to transport droplets to the middle and lower parts of the crop.
3. The field test showed that the droplet deposition and coverage decreased gradually along the range direction, and the top layer had the highest deposition and coverage across the canopy. The upper canopy of the range of 0 to 12 metres range demonstrated a greater extent of coverage and deposition. However, there was no significant enhancement in the lower canopy, indicating that the multi-heads hydraulic nozzle has limited ability to penetrate this area. The peak deposition area expanded from 9 to 33 metres in the lower canopy, with an average value of 3.77 μg cm^-2^. This indicated that as the range extended, deposition in the lower and middle crop layers dispersed further, reflecting the advantages of a downward-pressure wind field.

## Data availability statement

The original contributions presented in the study are included in the article/supplementary material. Further inquiries can be directed to the corresponding author.

## Author contributions

YM: Conceptualization, Methodology, Validation, Writing – original draft, Writing – review & editing. XC: Formal Analysis, Methodology, Validation, Writing – review & editing. YG: Conceptualization, Funding acquisition, Project administration, Resources, Supervision, Writing – review & editing. DL: Investigation, Methodology, Writing – review & editing. JC: Resources, Writing – review & editing. GW: Data curation, Visualization, Writing – review & editing. XZ: Data curation, Investigation, Writing – review & editing.
